# Improved clinical outcome after medial open-wedge osteotomy despite cartilage lesions in the lateral compartment

**DOI:** 10.1371/journal.pone.0224080

**Published:** 2019-10-24

**Authors:** Lisa Hohloch, Suchung Kim, Helge Eberbach, Kaywan Izadpanah, Julian Mehl, Philipp Niemeyer, Norbert P. Südkamp, Gerrit Bode

**Affiliations:** 1 Department of Orthopedics and Trauma Surgery, Medical Center - Albert-Ludwigs-University of Freiburg, Faculty of Medicine, Albert-Ludwigs-University of Freiburg, Freiburg, Germany; 2 Center for Musculoskeletal Surgery, Charité-Universitaetsmedizin Berlin, Berlin, Germany; 3 Department of Orthopaedic Sports Medicine, Hospital Rechts der Isar, Technical University of Munich, Munich, Germany; 4 OCM Orthopädische Chirurgie München, Munich, Germany; University of California, Irvine, UNITED STATES

## Abstract

High tibial medial open-wedge osteotomy (HTO) is an established treatment option for cartilage lesions in the medial compartment. It was this study’s aim to evaluate the effect of asymptomatic single or kissing lesions in the lateral compartment on functional outcome after medial open-wedge osteotomy. A total of 156 patients were enrolled in this retrospective study. All patients underwent HTO due to a varus deformity and a symptomatic cartilage lesion or osteoarthritis in the medial compartment. We acquired preoperative Lysholm and VAS Scores. Each open-wedge osteotomy was preceded by diagnostic arthroscopy to ensure the compartments were thoroughly documented and diagnosed. Cartilage lesions in the lateral compartment were evaluated, and three groups created according to their individual characteristics: group A (no cartilage lesion, n = 119), group B (single cartilage lesion, n = 16) and group C (kissing lesions, n = 21). Cartilage lesions were graded according to the Outerbridge classification, The functional postoperative outcome was determined by relying on several parameters (VAS Score, Lysholm, KOOS, WOMAC Score). Pre- and postoperative long-leg axis views were analyzed via special planning software (mediCAD, Hectec GmbH Germany). Mean follow-up was at 69.0 ± 30.3 months after surgery (range 22 to 121 months). There were no significant differences between the three groups in the correction angle chosen (p = 0.16). Regarding the outcome parameters, group A attained the best results in the WOMACpain Score (p = 0.03) and WOMACfunction Score (p = 0.05). A higher Outerbridge-Score of cartilage lesions in the lateral compartment was associated with a higher (i.e., worse) WOMACpain Score (p = 0.018) and WOMACfunction Score (p = 0.033). In all the groups (A, B, and C), HTO led to a significant improvement in the Lysholm Score (p < 0.001) and to a decrease in pain level (VAS Score; p < 0.001). Conclusion: Valgus high tibial osteotomy leads to reduced pain and improved functional outcome scores, even in patients with pre-existing asymptomatic single or corresponding cartilage lesions in the lateral compartment. In case of severe cartilage lesions in the lateral compartment, surgeons should consider that clinical outcome worsens depending on the Outerbridge Score.

## Introduction

High tibial osteotomy (HTO) has become an established and reliable treatment option for patients suffering from a varus deformity and symptomatic medial osteoarthritis (OA) or a cartilage lesion in the medial compartment.[1] In a prior study, 96% of the patients examined required no conversion to total knee arthroplasty after a mean follow-up of 60.5 months after therapy with HTO[2]. Recently, many factors concerning planning and technique of the valgus HTO have been established: prior to an HTO, the extent of correction should be meticulously planned according to the indications for surgery [3,4]. Patients undergoing HTO for medial osteoarthritis should also undergo early weight-bearing follow-up therapy [5]. The effect of standard correction and over-correction on the lateral compartment was well investigated by Madry and Ziegler et al. in a sheep model[6–9]. Mina et al. demonstrated biomechanically that neutralization or a slight valgus correction of the leg axis leads to an equal distribution of load between the medial and lateral compartment.[10] A further valgus correction leads to complete unloading of the medial compartment and maximum load on the lateral compartment[10]. Pain and significant cartilage lesions in the lateral compartment have traditionally been considered a contraindication to valgus HTO[1,11]. Nonetheless, a worsening of lateral compartment cartilage damage in arthroscopic or MRI controls was not observed after valgus HTO, nor were significant morphologic changes detected in the lateral compartment [6,7,11–13]. To our knowledge, there has been no clinical evaluation of patients who have undergone valgus HTO despite the intra-operative detection of concomitant non-symptomatic cartilage lesions in the lateral compartment.

This study aimed to evaluate the effects of valgus HTO on patients with pre-existing cartilage damage in the lateral compartment. We hypothesized that a pathology in the lateral compartment would lead to an inferior clinical result of valgus-HTO indicated in case of unicompartimental medial gonarthritis (with osteoarthritic radiologic markers such as osteophytes, joint space narrowing, etc.) or symptomatic focal cartilage defects in the medial compartment or to an inferior clinical outcome.

## Materials and methods

### IRB approval

Freiburg University’s Ethics Committee approved this study (ID 118/14). All patients took part in the study voluntarily and signed an informed consent form in accordance with the Declaration of Helsinki.

This study was designed as a case series. From 2003 to 2013, a total of 205 patients with a varus deformity of their lower extremity of at least 2° and symptomatic OA or symptomatic focal cartilage damage in the medial compartment underwent a biplanar valgus HTO in open-wedge technique. Inclusion and exclusion criteria followed HTO indications [1]. Our inclusion criteria were thus unicompartimental medial gonarthritis (with osteoarthritic radiologic markers such as osteophytes, joint space narrowing, etc.) or a focal cartilage lesion in the medial compartment with an underlying varus deformity. Patients were not over 65 years of age, and the absolute range-of-motion was at least at a flexion/extension of 120–0°. Extension deficits were integrated in the preoperative planning.[1] Exclusion criteria were a significantly restricted flexion, as well as patients suffering from inflammatory arthropathy, extensive loss (> 2/3 of its surface) or absence of the lateral meniscus or high-grade ligamentous instabilities or severe general OA including the lateral and patellofemoral compartment. Patients were asked prior to surgery about pain in the lateral compartment. If they felt pain, they did not qualify for medial open-wedge osteotomy. Mean follow-up lasted 69.0 ± 30.3 months (range 22 to 121 months); data acquisition took place in December 2013. A total of 156 patients were included in the study. 23.9% of all patients (n = 49) were lost to follow-up because of: they wished to withdraw from the study (n = 9), postoperative radiologic documentation of the long-leg axis was incomplete or unavailable (n = 14), due to undercorrection (n = 18), or because of a change in address (n = 8).

Preoperative data included the Lysholm Score and Visual Analogue Scale (VAS) for estimating pain levels, as well as the extent of varus alignment (in degrees).

The extent of correction was measured at the time of follow-up using planning software (mediCAD, Hectec GmbH, Germany). Intra-articular structures of all compartments were examined during diagnostic arthroscopy to detect lesions to be treated during surgery, and to confirm the indication for HTO. Cartilage lesions in the medial, lateral, and patellofemoral compartments were registered and classified according to the Outerbridge classification.[14]

Patients were separated into three groups according to the presence of cartilage lesions in the lateral compartment and according to the lesion’s location. Group A revealed no lesion in the lateral compartment. Group B was diagnosed with focal cartilage damage to the lateral femoral condyle (LFC) or lateral tibial plateau (LTP). Group C presented a kissing lesion (femoral and tibial focal cartilage lesion) in the lateral compartment.

The HTO technique was chosen in accordance with the AO Expert group’s technique [15]. In all cases, an ascending biplanar step osteotomy was performed without the use of additional bone grafts, and an internal plate fixator was used to stabilize the osteotomy (TomoFix^™^ system, Synthes, Solothurn, Switzerland). The extent of correction planned preoperatively was intraoperatively secured by a navigation system (Orthopilot^™^; Aesculap Co. Tuttlingen, Germany; Software: Orthopilot software for HTO). Our postoperative rehabilitation protocol was adjusted according to the indication for surgery and followed protocols already published [2,16].

Briefly summarized, all patients were mobilized on the first post-operative day. Continuous passive motion was recommended to all patients after HTO from day 1 postoperatively for 6 weeks for up to 4 h per day. Limited weight-bearing was recommended for 2–3 weeks after HTO. Thereafter, weight-bearing was increased stepwise to full weight-bearing by week 4 after surgery. Only patients with additional cartilage repair procedures underwent a longer partial weight-bearing period for 6 weeks, followed by an increase to full weight-bearing within 10 days. Once full weight-bearing was possible, full-leg radiographs were taken to analyze the post-operative weight-bearing axis of the affected lower limb.

Functional outcome was measured at final follow-up via standardized scores (Lysholm Score[17]; Knee Injury an Osteoarthritis Outcome Score (KOOS)[18] with its subscores KOOS pain, KOOS symptoms, KOOS adl, KOOS sports, KOOS QOL, KOOS 4; Western Ontario and McMaster Universities Osteoarthritis Index (WOMAC Score)[19] with its subscores WOMAC pain, WOMAC stiffness and WOMAC function. Pain levels were estimated by the VAS.

We acquired an anterioposterior weight-bearing long axis view six weeks after surgery to ensure that the result planned preoperatively had indeed been achieved. Postoperative tibial slope correction was also assessed via a lateral view. Any complications leading to revision surgery were recorded.

For statistical analysis, data was collected in Microsoft Excel XP (Microsoft, Redmond Washington) and detailed evaluations done via SPSS Statistics 24.0 (IBM Corp., Armonk, USA). Analysis included an explorative data analysis with continuous variables presented as mean ± standard deviation (SD) and categorical variables described as frequencies and percentages. ANOVA was used to compare normally distributed data and the Kruskal-Wallis and Mann-Whitney-U test for non-normally distributed data. The Chi-Squared Test was used to compare frequencies. The Pearson’s correlation coefficient, respectively the Spearman’s rho were used to identify correlations. A p-value of < 0.05 was considered statistically significant.

The article processing charge was funded by the German Research Foundation (DFG) and the University of Freiburg in the funding programme Open Access Publishing.

## Results

### Characteristics of the study cohort

Of our cohort of 156 patients, 41 patients were female, 115 patients were male. Group A consisted of 119 patients, group B of 16 patients, and group C of 21 patients. The Outerbridge classification of lesions in the lateral compartment is illustrated in Table 1. Predominantly superficial lesions were observed (Outerbridge grade 1 or 2) in groups B and C. None of the cartilage lesions in the lateral compartment underwent cartilage surgery as they were pre-operatively non-symptomatic. In some patients of group A, B and C patellofemoral cartilage lesions were present, predominantly in a lesser degree of severity (Table 2). There were no significant differences between groups in the duration of follow-up, BMI, pre-operative VAS, Lysholm score or duration of symptoms until surgery. Group C patients were significantly older than group A’s and B’s. Group C presented a significantly larger preoperative varus deformity than group A. Group B contained a significantly higher proportion of female patients than group A and group C.

**Table 1:** Intra-operative arthroscopic classification (Outerbridge Score[14]) of cartilage lesions in the lateral compartment.

Outerbridge Score	Description	Group B (n)	Group C (n)
1	Superficial lesions, fissures and cracks, soft indentation	2	6
2	Fraying, lesions extending down to < 50% of cartilage depth	11	13
3	Partial loss of cartilage thickness, cartilage defects extending down to > 50% of cartilage depth as well as down to calcified layer	2	1
4	Complete loss of cartilage thickness, bone only	1	1

**Table 2:** Demographic and surgical data of group A, B and C at the time of surgery with means and standard deviations. Significant differences are highlighted.

	Group A	Group B	Group C
Duration of follow-up (in months)	66.44 ± 28.32	70.15 ± 28.09	69.47 ± 25.09*
BMI (kg/m^2^)	26.75 ± 4.17	28.47 ± 5.47	27.71 ± 4.38
Age (in years)	42.61 ± 10.14	47.12 ± 5.52	52.15 ± 9.14
Lysholm score	47.18 ± 19.32	35.54 ± 15.29	41.71 ± 21.09
Pre-operative VAS	7.00 ± 2.00	7.38 ±1.12	7.42 ± 1.80
Duration of symptoms until surgery (in months)	21.86 ± 19.51	22.46 ± 17.42	24.32 ± 18.61
Pre-operative varus deformitiy (in °)	5.55 ± 2.39	7.01 ± 3.42	7.37 ± 3.07*
Ratio male: female patients	91: 28	8: 8*	16: 5
Cartilage lesions in the lateral tibial plateau (n, mean Outerbridge Score)	-	10 (mean Outerbridge score 2.0 ± 0.5, range 1 to 3)	21 (mean Outerbridge Score 1.9 ± 0.7, range 1 to 4)
Cartilage lesions in the lateral femoral condyle (n, Outerbridge Score)	-	6 (mean Outerbridge score 2.3 ± 1.0, range 1 to 4)	
Cartilage lesions in the patellofemoral compartment (n, mean Outerbridge Score)	23 corresponding, 18 patellar, 11 trochlear cartilage lesions (1.11 ± 1.37, range 0 to 4)	2 corresponding, 7 patellar, 2 trochlear cartilage lesions (1.81 ± 1.42, range 0 to 4)	11 corresponding, 3 patellar, 0 trochlear cartilage lesions (1.76 ± 1.50, range 0 to 4)

*, p-value.

### Postoperative radiological analysis

Our analysis of all antero-posterior weight-bearing long axis views that are displayed in Table 3 showed that patients in the different groups did not differ to a significant extent in the postoperative degree of valgus (p = 0.66), postoperative valgus position on the tibial plateau (p = 0.98), or in the correction angle (p = 0.16). Tibial slope changed minimally from a mean of 7.5° ± 3.3° (range 0° to 15.10°) preoperatively to 9.3° ± 3.9° (range 0.84° to 20.70°) postoperatively.

**Table 3:** Postoperative axis correction.

Mean	Group A	Group B	Group C
Postoperative valgus position (in % of tibial plateau)	59.6 ± 8.4	62.1 ± 8.8	59.7 ± 5.5
Postoperative valgus (in °)	2.7 ± 1.7	3.1 ± 1.9	2.4 ± 1.5
Correction angle (in°)	8.0 ± 2.9	9.1 ± 4.7	9.8 ± 3.8

### Functional outcome and correlations

WOMACpain (p = 0.03) and WOMACfunction Scores (p = 0.05) revealed a significantly better functional outcome in group A (WOMAC pain: 60.1 ± 38.5, WOMACfunction: 63.6 ± 36.8) than in group B (WOMACpain: 74.7 ± 22.5, WOMACfunction 78.5 ± 20.2) or C (WOMACpain: 79.0 ± 19.9, WOMACfunction 78.9 ± 19.1).

**Fig 1:**
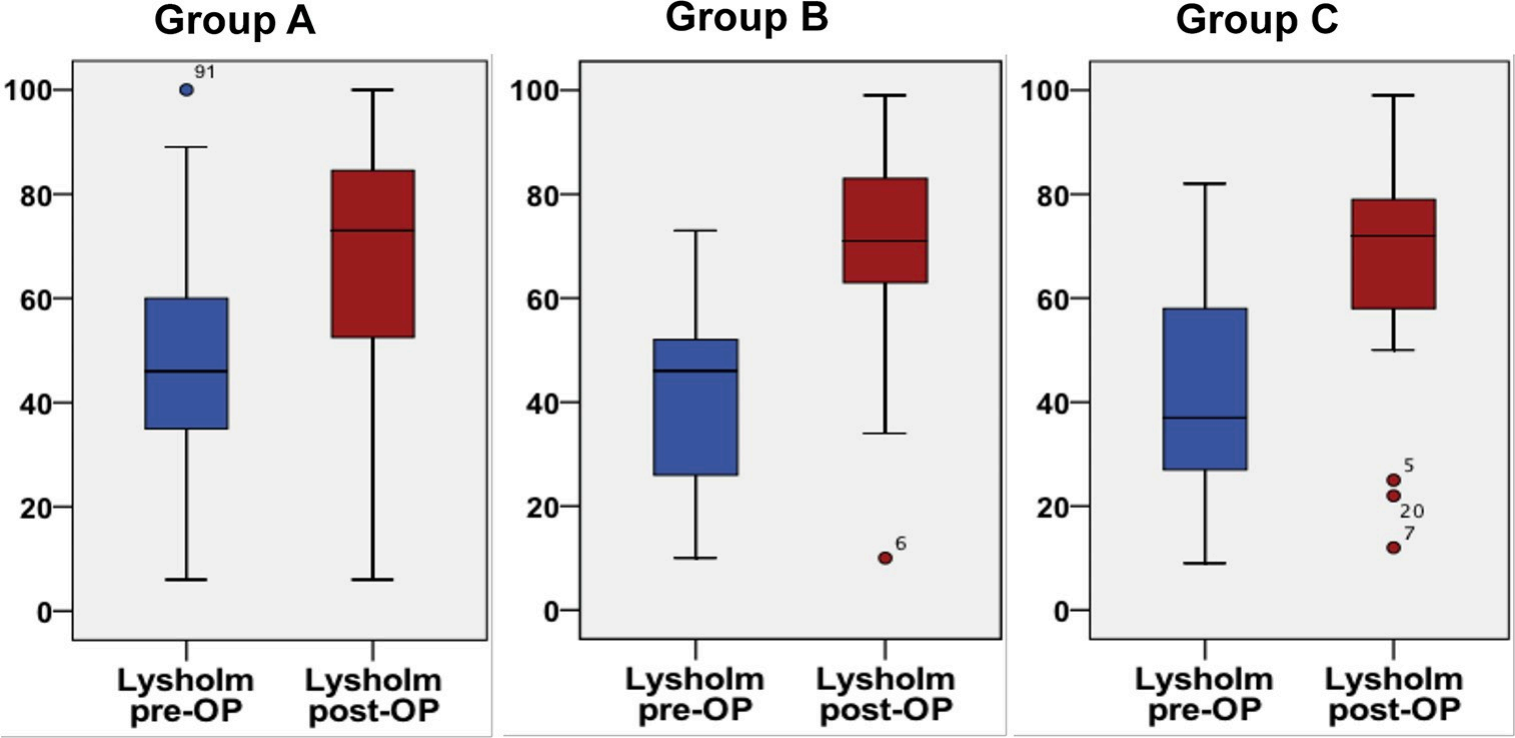
Comparison of preoperative to postoperative Lysholm scores. Groups A (p < 0.001), B (p < 0.001), and C (p < 0.001) exhibited a significantly improved clinical outcome.

**Fig 2:**
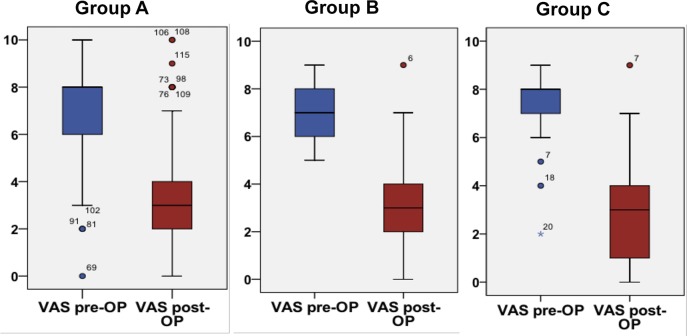
Comparison of preoperative to post-operative pain levels (VAS). Groups A (p < 0.001), B (p < 0.001), and C (p < 0.001) revealed a significant reduction in pain.

Biplanar valgus HTO led to a significantly reduced pain level (VAS) and to a significantly higher Lysholm Score in all three groups compared to preoperative values (Figs 1 and 2).

A higher Outerbridge score of the lateral compartment was associated with significantly higher WOMACpain (LTP: p = 0.015, LFC: p = 0.018) and WOMACfunction Scores (LTP: p = 0.022, LFC: p = 0.033). Groups B and C demonstrated no significant correlation between the extent of correction or postoperative valgus angle and final functional outcome scores.

Revision surgery was performed in group A in 10 of 119 patients, in group B in 3 of 16 patients and in group C in 1 of 20 patients. We noted significant differences between groups B and C (p = 0.013). None of the revision interventions was due to pain in the lateral compartment. Two patients required an early postoperative intervention due to significant overcorrection in the valgus as revealed in postoperative X-rays (5.5°, respectively 6.8° of valgus) even though intraoperative navigation was used. The other indications for revision surgery, their characteristics and their post-operative outcomes are displayed in Table 4.

**Table 4:** Characteristics and outcome of the 14 patients who required revision surgery.

Patient (gender)	Reason for revision	Characteristics	Outcome parameters
1, female	Infection	Age 30.2 years BMI 21.1 (kg/m^2^) Non-smoker	VASpost: 1 (pre: 6) Lysholmpost: 79 (pre: 71) KOOS4: 78.4
2, female	Popliteal aneurysm (necessitating Vascular surgery)	Age 50.4 years BMI 29.1 (kg/m^2^) Smoker	VASpost: 2 (pre: 10) Lysholmpost: 79 (pre: 71) KOOS4: 83.53
3, male	Hematoma Wound healing disturbance	Age 37.2 years BMI 27.8 (kg/m^2^) Smoker	VASpost: 1 (pre: 7) Lysholmpost: 94 (pre: 50) KOOS4: 81.45
4, male	Non-union	Age 48.2 years BMI 30.9 (kg/m^2^) Non-Smoker	VASpost: 2 (pre: 9) Lysholmpost: 99 (pre: 57) KOOS4: 77.23
5, female	Wound healing disturbance	Age 54.8 years BMI 22.1 (kg/m^2^) Non-Smoker	VASpost: 3 (pre: 7) Lysholmpost: 65 (pre: 30) KOOS4: 53.73
6, female	Infection	Age 58.5 years BMI 30.9 (kg/m^2^) Non-Smoker	VASpost: 1 (pre: 8) Lysholmpost: 94 (pre: 21) KOOS4: 60.97
7, male	Non-union	Age 50.7 years BMI 32.5 (kg/m^2^) Non-Smoker	VASpost: 1 (pre: 8) Lysholmpost: 79 (pre: 32) KOOS4: 68.89
8, male	Non-union	Age 54.0 years BMI 29.1 (kg/m^2^) Non-Smoker	VASpost: 5 (pre: 8) Lysholmpost: 55 (pre: 49) KOOS4: 56.35
9, male	Wound healing disturbance	Age 34.8 years BMI 27.7 (kg/m^2^) Non-Smoker	VASpost: 0 (pre: 5) Lysholmpost: 100 (pre: 60) KOOS4: 88.77
10, female	Overcorrection	Age 47.5 years BMI 37.9 (kg/m^2^) Non-Smoker	VASpost: 0 (pre: 6) Lysholmpost: 99 (pre: 40) KOOS4: 89.84
11, male	Wound healing disturbance	Age 56.9 years BMI 27.2 (kg/m^2^) Non-Smoker	VASpost: 6 (pre: 6) Lysholmpost: 56 (pre: 40) KOOS4: 74.2
12, female	Overcorrection	Age 37.4 years BMI 26.6 (kg/m^2^) Non-Smoker	VASpost: 3 (pre: 8) Lysholmpost: 53 (pre: 53) KOOS4: 74.16
13, male	Non-union	Age 33.0 years BMI 24.1 (kg/m^2^) Smoker	VASpost: 5 (pre: 10) Lysholmpost: 49 (pre: 44) KOOS4: 49.26
14, male	Wound healing disturbance	Age 48.3 years BMI 28.7 (kg/m^2^) Smoker	VASpost: 1 (pre: 7) Lysholmpost: 74 (pre: 51) KOOS4: 63.81

## Discussion

The most important findings of the present study are that HTO leads to a significant postoperative improvement in functional outcome scores even if asymptomatic subtotal single or kissing cartilage lesions in the lateral compartment are present. Most of our study patients (76.3%) who underwent valgus HTO exhibited no cartilage lesions in the lateral compartment. A valgus HTO was carried out in 37 patients, although their diagnostic arthroscopy showed cartilage damage in the lateral compartment. We even diagnosed “kissing lesions” in 21 patients. The lesions located in the lateral compartment were mainly superficial lesions of Outerbridge grade 1 or 2 (n = 32), severe lesions of Outerbridge grade 3 (n = 3) or even 4 (n = 2) were very seldom diagnosed in the lateral compartment. No patient revealed any clinical signs of damage in the lateral compartment. Patients with kissing lesions were significantly older and had a larger varus deformity than patients in group A or B. We detected no significant differences between the three groups concerning the postoperative axis. The WOMACpain and WOMACfunction Scores revealed that group A’s functional outcome was superior to those of group B and C and a higher Outerbridge-Score of cartilage lesions in the lateral compartment of group B and C was associated with a higher WOMACpain Score and WOMACfunction Score. Nonetheless, comparison of most of the outcome scores (VAS, Lysholm, KOOS subscores) revealed no significant group differences. Group A, B and C patients enjoyed a significant benefit from their valgus HTO, which significantly reduced their pain (VAS score) and significantly increased their Lysholm scores. No patient had to undergo revision surgery for pain in the lateral compartment. Valgus HTO is a well-established treatment option for pathologies in the medial compartment of the knee, even in cases involving only a slight varus deviation of the leg axis[16]. It has traditionally been recommended that patients with significant cartilage lesions in the lateral compartment should not undergo HTO[1]. The effects of open-wedge HTO on the lateral compartment have been biomechanically and histologically evaluated [6–8,10,13], but no thorough clinical evaluation of patients who have undergone valgus HTO (although cartilage lesions in the lateral compartment had been diagnosed arthroscopically) has been carried out to date. Mina et al. concluded that a postoperative valgus angle of 0 to 4° sufficed to achieve an approximately equal load distribution between the medial and lateral compartment. A valgus measuring 6° to 10° led to a load shift towards the lateral compartment. The three groups in this study revealed mean postoperative valgus angles ranging from 2.4° to 3.1°. We were therefore able to avoid an additional load on the lateral compartment by the valgus HTO. There was thus no correlation between the extent of correction or postoperative valgus angle and final functional outcome scores in groups B and C. In a sheep model, Madry et al. demonstrated a correction to 4.5° valgus did not lead to morphological alterations and differences in the DNA and proteoglycan content of the lateral menisci after a 6-week follow-up period. An overcorrection to 9,5° of valgus significantly reduced cell numbers in the middle third of the red-red zone of the lateral menisci (reduced DNA contents compared to control knees without HTO). Even in this group, there were no detectable morphological alterations in the mid-term[6]. A sheep model can be used to simulate interventions in the human knee, although some differences need to be accepted[20]. Ziegler et al. used a sheep model to examine the effects of a valgus HTO on cartilage in the lateral compartment[13]. Similar to human knees[21], sheep knees demonstrate a thicker cartilage layer in the central region (sheep: about 4.6 fold thicker, human beings: about 2-fold thicker) than in the peripheral region that the lateral meniscus covers. Histologically, safranin O is more intense in the central region, thus indicating a higher concentration of proteoglycans in this region. Osteoarthritic changes predominantly take place in the central region. However, six months after surgery, neither a correction to 4.5° of valgus nor overcorrection to 9.5° of valgus led to macroscopic changes in the lateral compartment[13]. In this study, correction angles larger than 3° were completely avoided in groups B and C with concomitant (arthroscopically diagnosed) cartilage lesions in the lateral compartment.

### Study limitations

First of all, our study carries all the limitations associated with a retrospective investigation. Large patient cohorts are necessary to detect associations. This clinical study experienced follow-up loss of 23.9%. Nonetheless, 156 patients were included in the follow-up examinations—quite a high number compared to similar studies [16,22,23]. Considering that the subgroup we examined represents a highly selected subgroup, we believe our patient numbers in each group, the study’s design and follow-up seem appropriate and reasonable despite the lack of a power analysis.

A further potential limitation is the characteristics of groups A, B, and C before surgery. Although they were largely homogeneous, the group C patients were significantly older than those in group A and B, and the sex ratio of group B differed from groups A and C—factors that could have influenced the final outcome. Nonetheless, as this study examines borderline indications of the valgus HTO, we had to tolerate this inhomogeneity.

Additionally only Lysholm scores were filled out pre- and postoperatively while KOOS and WOMAC—Scores were only obtained postoperatively.

Unfortunately, we were unable to arrive at a correlation of outcome parameters with the size of cartilage lesions in the lateral compartment, as defect sizes in the lateral compartment were seldom measured or documented in surgery reports. Focus during surgery was on the size of cartilage lesions in the medial compartment. An examination of these scientific issues would be an interesting subject of further prospective studies.

### Conclusion

HTO leads to reduced pain and improved functional outcome scores even in patients with pre-existing asymptomatic cartilage lesions in the lateral compartment. Thus, the presence of cartilage lesions in the lateral compartment does not necessarily constitute a contraindication in HTO patients.
